# Insomnia and psychological disintegration: Evidence from a transdiagnostic network analysis

**DOI:** 10.1371/journal.pone.0354243

**Published:** 2026-07-23

**Authors:** Niloufar Moohebat, Farhad Khormaei

**Affiliations:** Department of Educational Psychology, Shiraz University, Shiraz, Iran; Johns Hopkins: Johns Hopkins University, UNITED STATES OF AMERICA

## Abstract

Insomnia is commonly conceptualized in terms of hyperarousal or symptom amplification; however, less attention has been paid to the structural organization and integration of psychological processes underlying sleep disturbance. This study examined whether insomnia is associated with reduced integration among psychological domains rather than elevated symptom activation alone. A comparative network analysis was conducted on 27 transdiagnostic psychological markers in a sample of 391 Iranian university students (169 poor sleepers, 222 good sleepers). Gaussian graphical models were estimated using graphical LASSO regularization with EBIC model selection. Network structure, expected influence, and node predictability were examined, and Network Comparison Tests were used to assess structural and global strength differences between groups. Compared with good sleepers, poor sleepers exhibited markedly sparser network structures, a greater number of isolated nodes, and substantially lower node predictability. No significant differences were observed in global network strength between groups. Several cognitive variables tended to show higher centrality in good sleepers, suggesting context-dependent roles within more integrated psychological systems. These findings indicate that insomnia may be characterized by reduced integration among psychological processes rather than uniformly elevated symptom activation, reflecting a systems-level characteristic of psychological functioning associated with sleep disturbance.

## Introduction

Insomnia is highly prevalent among university students, a population exposed to substantial academic, social, and circadian challenges. Epidemiological studies indicate that between 35% and 68% of students report clinically significant sleep disturbances, a rate markedly higher than that observed in the general adult population [1,2]. Beyond sleep complaints, insomnia has been consistently associated with a wide range of adverse outcomes, including depression, anxiety, cognitive impairment, and increased risk for suicidal ideation [3]. Despite the availability of evidence-based treatments such as cognitive-behavioral therapy for insomnia (CBT-I), long-term outcomes remain modest, with relapse rates exceeding 30% [4,5]. These limitations suggest that existing models may not fully capture the psychological processes underlying persistent sleep disturbance.

Prevailing theoretical accounts of insomnia primarily emphasize hyperarousal, heightened emotional reactivity, or amplification of maladaptive cognitions [6,7]. According to these models, trait vulnerabilities combined with stressors lead to increased physiological and cognitive activation, which in turn disrupts sleep. While this framework has generated important insights, it does not fully explain why individuals exposed to similar stressors exhibit markedly different sleep outcomes, nor why reductions in specific symptoms do not consistently translate into sustained improvements in sleep quality.

Network approaches to psychopathology conceptualize mental disorders as emerging from patterns of interaction among psychological elements rather than from single latent causes [5]. Although recent network studies of insomnia have identified central symptoms such as rumination, worry, and negative affect [8,9,10,11], most investigations have focused on identifying highly connected nodes rather than examining the overall organization and integration of psychological domains.

Building on network theory [5], the present study considers the possibility that insomnia may be associated with alterations in the integration of psychological processes rather than uniformly elevated activation. Psychological integration refers to the degree to which cognitive, affective, personality, and regulatory elements are functionally connected and mutually constraining within a system. In more integrated networks, psychological processes may contextualize and regulate one another, whereas reduced integration may result in fragmentation, isolation of elements, and diminished predictability of psychological dynamics. Concepts from complex systems theory and psychological entropy further support the importance of integration for adaptive functioning [12,13].

The current study addresses this important gap by testing whether insomnia is associated with reduced psychological integration—characterized by sparser connectivity, more isolated nodes, and lower node predictability—across transdiagnostic domains including personality traits (providing stable regulatory contexts), emotional symptoms, cognitive distortions (whose functional impact is context-dependent), and emotion regulation strategies. From this perspective, cognitions commonly labeled as maladaptive may function differently depending on their network context. When embedded within coordinated systems involving planning, personality traits, or adaptive regulation strategies, such cognitions may serve constructive roles; when isolated, they may contribute to distress. This framework yields a testable prediction: individuals with insomnia will exhibit more fragmented psychological networks—characterized by sparser connectivity, isolated nodes, and lower node predictability—without necessarily showing greater overall network strength.

The present study tested this hypothesis using a comparative network analysis of 27 transdiagnostic psychological markers encompassing personality traits, emotional symptoms, cognitive distortions, and emotion regulation strategies. Networks were estimated separately for poor and good sleepers in a sample of Iranian university students, an underrepresented but high-risk population in sleep research. By examining differences in network structure, connectivity, expected influence, and node predictability, this study aimed to determine whether insomnia is associated with disruptions in psychological integration rather than generalized increases in symptom activation.

## Methods

### Ethics approval and consent to participate

The study was approved by the Research Ethics Committee of the Faculty of Psychology and Educational Sciences, Shiraz University (Approval ID: IR.US.PSYEDU.REC.1403.129; approved on March 12, 2025). All procedures were conducted in accordance with the ethical standards of the institutional research committee and the 1964 Declaration of Helsinki and its later amendments. Written informed consent was obtained from all participants prior to completing the questionnaires.

### Participants and procedure

A total of 391 undergraduate students (mean age = 21.5 ± 2.1 years; 58% female) from Shiraz University participated in this study. Participants were enrolled in various undergraduate programs (e.g., humanities, engineering, basic sciences) and were in their first to fourth year of study. Participants were recruited using convenience sampling between June 1, 2025 and October 10, 2025. Although this sampling method is practical, it may introduce selection bias and limit the generalizability of the findings. Written informed consent was obtained from all participants prior to completing the questionnaires. All participants were adults (aged ≥18 years); therefore, parental or guardian consent was not required.

Insomnia status was assessed using the Persian version of the Pittsburgh Sleep Quality Index (PSQI-P) [14]. The cutoff score (≥6) was based on the validated Persian version of the Pittsburgh Sleep Quality Index, which demonstrated optimal sensitivity and specificity for distinguishing poor and good sleepers. Participants with PSQI scores ≥6 were classified as poor sleepers (n = 169), whereas those with scores ≤5 were classified as good sleepers (n = 222). The groups did not differ significantly in age, sex, or academic year (all p > 0.05).

### Measures

All instruments were validated Persian versions. Detailed psychometric properties including reliability coefficients and validity evidence are provided in S1 Table.

Sleep quality was assessed using the Persian version of the Pittsburgh Sleep Quality Index (PSQI-P; Cronbach’s α = 0.76) [14]. The bed-partner item and open-ended item 5j were omitted in accordance with prior validation studies.

Personality traits were measured using a 15-item short form of the Big Five Inventory, which has demonstrated acceptable psychometric properties in Persian samples [15,16].

Psychological distress was assessed using the Depression Anxiety Stress Scales (DASS-21; Persian version with strong reliability and validity: α = 0.79–0.93) [17,18].

Cognitive emotion regulation strategies were measured using the Cognitive Emotion Regulation Questionnaire (CERQ-P; α = 0.83) [19].

Cognitive distortions were assessed using the Cognitive Distortions Questionnaire (CDQ; α = 0.84) [20].

### Data analysis

#### Network nodes.

Twenty-seven nodes spanning four domains—personality traits, emotional symptoms, cognitive distortions, and emotion regulation strategies—were included in the analyses (S2 Table).

#### Network estimation and robustness.

Gaussian graphical models were estimated separately for poor and good sleepers using the R package bootnet [21] with graphical LASSO regularization and EBIC model selection (γ = 0.5) [22]. Expected influence centrality [23] and node predictability (R^2^) [24] were calculated. Expected influence centrality was selected over strength centrality because it preserves the direction of associations, which is particularly relevant in networks containing both positive and negative edges. Network stability and accuracy were assessed using non-parametric bootstrapping with 1,000 resamples.

#### Missing data.

Missing data were minimal (<1.5% across all items) and handled using listwise deletion, as is standard in psychological network analysis when data are missing completely at random (MCAR).

#### Network comparison.

The Network Comparison Test (NCT) with 5,000 permutations was used to examine differences in network structure, global strength, individual edge weights, and node-wise expected influence between groups [25].

#### Software.

All analyses were conducted in R version 4.3.2 using the qgraph [26], bootnet [21], and NetworkComparisonTest [25] packages.

## Results

### Baseline characteristics

Demographic characteristics of the two groups are presented in Table 1. Poor and good sleepers did not differ significantly in age, sex distribution, or academic year (all p > 0.05).

**Table 1:** Demographic characteristics of poor and good sleepers.

Variable	Poor Sleepers (n = 169)	Good Sleepers (n = 222)	p-value
Age (M ± SD)	21.03 ± 2.32	20.74 ± 2.91	0.82
Female, n (%)	85 (50.3)	102 (45.9)	0.36
Male, n (%)	84 (49.7)	120 (54.1)	0.36
Academic year (range)	1-4	1-4	>0.05

The two groups did not differ significantly in any demographic characteristics.

Descriptive statistics (means and standard deviations) for the 27 psychological variables are presented in S3 Table.

### Network structure

**Fig 1:**
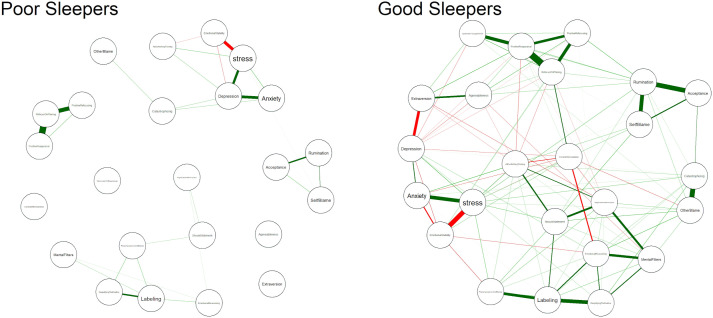
Comparative psychological networks. Partial correlation networks for poor sleepers (PS) and good sleepers (GS). Green edges represent positive partial correlations and red edges represent negative partial correlations. Node size reflects expected influence centrality.

The network estimated for poor sleepers was extremely sparse (density = 5.1%; 18 of 351 possible edges) and contained twelve isolated nodes. In contrast, the good-sleeper network was substantially denser (density = 18.2%; 64 edges) and contained no isolated nodes (Fig 1).

### Network accuracy and stability

**Fig 2:**
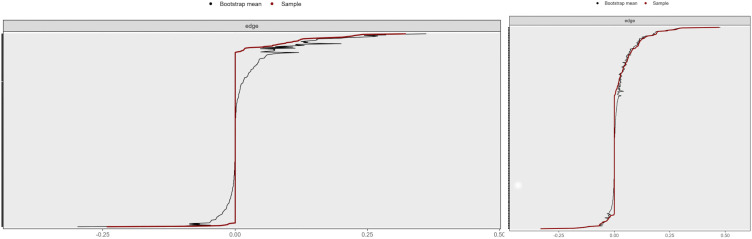
Non-parametric bootstrapping results. Bootstrapped confidence intervals for edge weights in the poor-sleeper (PS) and good-sleeper (GS) networks.

Bootstrap analyses indicated high edge-weight accuracy in both networks. The poor-sleeper network showed a narrow distribution of edge weights clustered near zero, whereas the good-sleeper network exhibited a wider distribution indicative of stronger connectivity (Fig 2). Centrality stability was excellent in both groups, with correlation stability coefficients for expected influence of 0.75, exceeding recommended thresholds [21].

### Global network properties

The Network Comparison Test (NCT) revealed a significant difference in overall network structure between groups (M = 6.96, p = 0.004) but no significant difference in global strength (M = 0.19, p = 0.712; Table 2).

**Table 2:** Network Comparison Test results.

Test	Statistic	*p*	Significant
Network Structure	6.96	.004	Yes
Global Strength	0.19	.712	No

*p* < .05 in bold. NCT with 5,000 permutations.

### Node centrality

**Fig 3:**
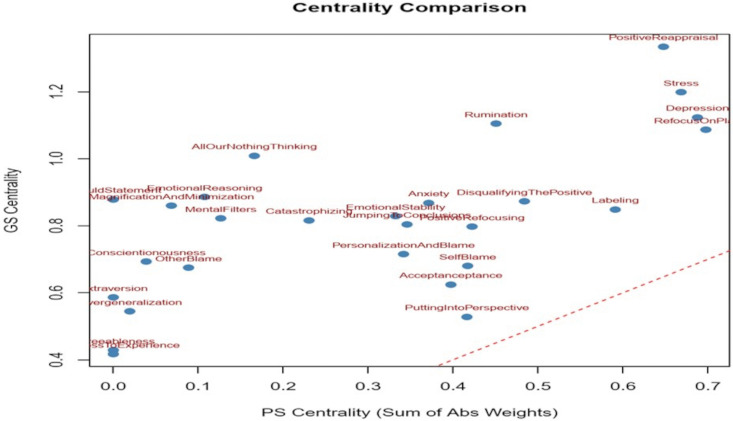
Differences in expected influence centrality. Points above the diagonal indicate higher expected influence centrality in good sleepers compared with poor sleepers.

All cognitive distortions demonstrated descriptively higher expected influence in the good-sleeper network compared to the poor-sleeper network. The largest differences were observed for should statements, all-or-nothing thinking, magnification and minimization, emotional reasoning, and mental filtering (Fig 3 and Table 3). However, none of these differences remained statistically significant after false discovery rate (FDR) correction and should therefore be interpreted with caution.

**Table 3:** Top five centrality differences (PS − GS).

Node	PS Centrality	GS Centrality	Δ (PS − GS)
Should Statement	0.04	0.92	−0.88
All-or-Nothing Thinking	0.17	1.01	−0.84
Magnification & Minimization	0.08	0.87	−0.79
Emotional Reasoning	0.10	0.88	−0.78
Mental Filters	0.13	0.83	−0.70

Negative Δ values indicate higher centrality in good sleepers (GS). None of the differences survived FDR correction.

### Edge-level differences

**Fig 4:**
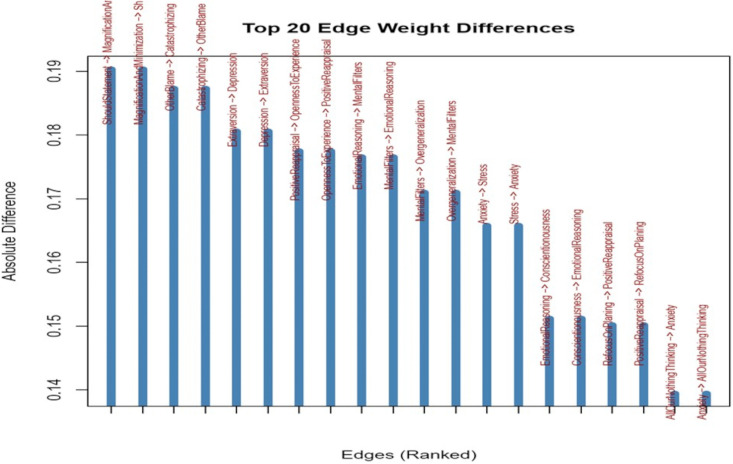
Top 20 absolute edge-weight differences between groups. Higher bars indicate greater divergence between groups. All displayed edges were stronger in the good-sleeper network.

Thirteen edges differed significantly between the poor-sleeper and good-sleeper networks at an uncorrected α = 0.05, with all edges being stronger in the good-sleeper network (Fig 4 and Table 4). Notable cross-domain connections included extraversion–depression and openness–positive reappraisal. However, none of these differences remained statistically significant after false discovery rate (FDR) correction for multiple comparisons.

**Table 4:** Top five edge-weight differences between poor sleepers (PS) and good sleepers (GS).

Edge	Δ (Abs)	*p (uncorrected)*
ShouldStatement ↔ Magnification	0.19	.041
OtherBlame ↔ Catastrophizing	0.19	.068
Extraversion ↔ Depression	0.18	.027
Openness ↔ PositiveReappraisal	0.18	.019
MentalFilters ↔ EmotionalReasoning	0.18	.043

All displayed edges were stronger in the good-sleeper network. p-values are uncorrected. None of the edge differences survived false discovery rate (FDR) correction.

### Node predictability

**Fig 5:**
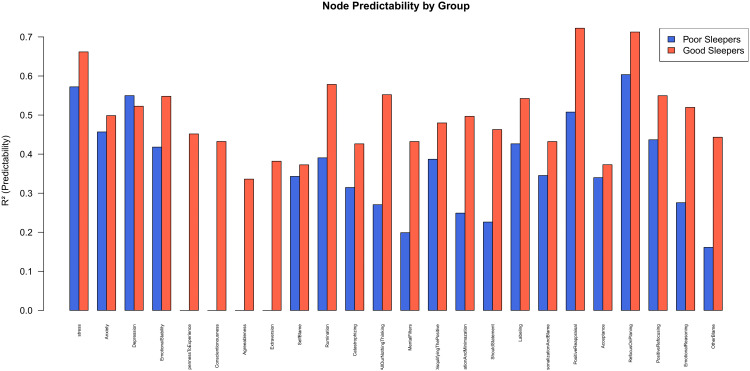
Node predictability by group. Node predictability (R^2^) values for poor sleepers and good sleepers, indicating the proportion of variance explained by neighboring nodes in each network.

Mean node predictability was markedly lower in poor sleepers (mean R^2^ = 0.02, range = 0.00–0.06) than in good sleepers (mean R^2^ = 0.41, range = 0.22–0.59). This pattern was consistent across nodes (Fig 5).

## Discussion

The present study examined whether insomnia is associated primarily with increased activation of psychological symptoms or with alterations in the organization and integration of psychological processes. Using a comparative network approach, the findings demonstrated that poor sleepers exhibited markedly sparser psychological networks, a greater number of isolated nodes, and substantially lower node predictability than good sleepers. Importantly, these differences emerged in the absence of significant differences in global network strength. Together, these results suggest that insomnia may be characterized less by uniformly elevated psychological activity and more by disruptions in the coordination and interdependence of psychological processes.

From the perspective of network psychopathology [5], mental disorders are understood as emergent properties of interacting psychological elements rather than as the direct expression of a single latent cause. Within this framework, psychological integration reflects the extent to which cognitive, affective, personality, and regulatory processes mutually influence and constrain one another. The highly fragmented networks observed among poor sleepers indicate a breakdown in this integrative organization, which may undermine the system’s capacity for adaptive regulation and increase vulnerability to dysregulated affect and cognition. These findings are consistent with recent comparative network studies of good and poor sleepers [10,11].

A notable feature of the poor-sleeper network was the presence of multiple isolated nodes spanning personality traits, cognitive distortions, and emotion regulation strategies. The isolation of broad personality traits such as openness, conscientiousness, and extraversion suggest that these enduring dispositions may be less functionally engaged in regulating emotional and cognitive processes in individuals with insomnia. Similarly, several cognitive distortions appeared isolated or weakly connected in the poor-sleeper network. These findings support a contextual interpretation in which the functional impact of cognitions depends on their embedding within broader psychological systems.

Node predictability provided further evidence of system-level differences between groups. The extremely low predictability observed in poor sleepers indicates reduced psychological coherence, whereas the substantially higher predictability in good sleepers suggests more organized and deterministic psychological dynamics.

The absence of differences in global network strength has important clinical implications. While interventions focused solely on symptom reduction remain valuable, approaches that promote psychological integration—such as Acceptance and Commitment Therapy [27]—may offer additional benefits for individuals with insomnia. However, this possibility requires direct testing in future intervention studies.

These psychological findings complement neurobiological accounts of insomnia that emphasize altered functional connectivity [28]. Future multimodal research combining network analysis with neuroimaging is warranted.

### Limitations

Several limitations should be acknowledged. The cross-sectional design precludes causal inferences regarding the temporal relationship between network disintegration and insomnia. Additionally, reliance on self-report measures may introduce shared method variance and reporting bias. The use of convenience sampling from undergraduate students at a single university (Shiraz University) limits the generalizability of the findings to other populations, educational levels, and cultural contexts. Future studies should employ longitudinal designs, objective sleep measures, and more diverse samples.

In summary, the present findings suggest that insomnia is associated with disruptions in the integration and coordination of psychological processes rather than generalized increases in symptom activation. Network-based measures of psychological integration and predictability may contribute to a more nuanced understanding of insomnia-related psychological functioning and may help inform future intervention research.

## Supporting information

S1 Table Psychometric properties of all measures used in the study (reliability and validity indices). (DOCX)

S2 Table Description of the 27 network nodes. (DOCX)

S3 Table Descriptive statistics (means and standard deviations) for study variables by group. (DOCX)
