# Supplementary material for: Insomnia and psychological disintegration: Evidence from a transdiagnostic network analysis
Source: PLoS One. 2026 Jul 23;21(7):e0354243. doi: 10.1371/journal.pone.0354243 (PMC13395365; doi:10.1371/journal.pone.0354243)
Supplement: S1 Table — (DOCX) [file pone.0354243.s001.docx]

**S1 Table. Psychometric properties of all measures used in the study (reliability and validity indices).**

| **Instrument** | **Subscale (network node)** | **k (items)** | **Cronbach's α reported in the manuscript** | **Validity evidence** |
| --- | --- | --- | --- | --- |
| **PSQI-P** | *(instrument total)* | 17 | 0.76 [10] | \|  \| \| --- \|  \| Persian validation study [10] \| \| --- \| |
| **BFI-15** | *(instrument total)* | 15 | Not reported in the manuscript | \|  \| \| --- \|  \| Persian validation studies [11,24] \| \| --- \| |
|  | Emotional Stability | 3 | — |  |
|  | Openness to Experience | 3 | — |  |
|  | Conscientiousness | 3 | — |  |
|  | Agreeableness | 3 | — |  |
|  | Extraversion | 3 | — |  |
| **DASS-21** | *(instrument total)* | 21 | 0.79–0.93 [12,23] | Persian validation studies [12,23] |
|  | Stress | 7 | — |  |
|  | Anxiety | 7 | — |  |
|  | Depression | 7 | — |  |
| **CDQ** | *(instrument total)* | 20 | 0.84 [26] | Validation study [26] |
|  | All-or-Nothing Thinking | 2 | — |  |
|  | Overgeneralization | 2 | — |  |
|  | Mental Filters | 2 | — |  |
|  | Disqualifying the Positive | 2 | — |  |
|  | Jumping to Conclusions | 2 | — |  |
|  | Magnification and Minimization | 2 | — |  |
|  | Emotional Reasoning | 2 | — |  |
|  | Should Statements | 2 | — |  |
|  | Labeling | 2 | — |  |
|  | Personalization and Blame | 2 | — |  |
| **CERQ-P** | *(instrument total)* | 36 | 0.83 [25] | Persian validation study [25] |
|  | Self-Blame | 4 | — |  |
|  | Rumination | 4 | — |  |
|  | Putting into Perspective | 4 | — |  |
|  | Catastrophizing | 4 | — |  |
|  | Positive Reappraisal | 4 | — |  |
|  | Acceptance | 4 | — |  |
|  | Refocus on Planning | 4 | — |  |
|  | Positive Refocusing | 4 | — |  |
|  | Other-Blame | 4 | — |  |

**Note. Cronbach's α values and validity evidence are reported as presented in the manuscript. Validity evidence refers to the validation studies cited in the manuscript. No additional validity coefficients are reported.**
