# Supplementary material for: Insomnia and psychological disintegration: Evidence from a transdiagnostic network analysis
Source: PLoS One. 2026 Jul 23;21(7):e0354243. doi: 10.1371/journal.pone.0354243 (PMC13395365; doi:10.1371/journal.pone.0354243)
Supplement: S2 Table — (DOCX) [file pone.0354243.s002.docx]

**S2 Table. Description of the 27 network nodes.**

| **Domain** | **Instrument** | **Node label** | **k items** |
| --- | --- | --- | --- |
| Personality traits | BFI-15 | Emotional Stability | 3 |
| Personality traits | BFI-15 | Openness to Experience | 3 |
| Personality traits | BFI-15 | Conscientiousness | 3 |
| Personality traits | BFI-15 | Agreeableness | 3 |
| Personality traits | BFI-15 | Extraversion | 3 |
| Emotional symptoms | DASS-21 | Stress | 7 |
| Emotional symptoms | DASS-21 | Anxiety | 7 |
| Emotional symptoms | DASS-21 | Depression | 7 |
| Cognitive distortions | CDQ | All-or-Nothing Thinking | 2 |
| Cognitive distortions | CDQ | Overgeneralization | 2 |
| Cognitive distortions | CDQ | Mental Filters | 2 |
| Cognitive distortions | CDQ | Disqualifying the Positive | 2 |
| Cognitive distortions | CDQ | Jumping to Conclusions | 2 |
| Cognitive distortions | CDQ | Magnification and Minimization | 2 |
| Cognitive distortions | CDQ | Emotional Reasoning | 2 |
| Cognitive distortions | CDQ | Should Statements | 2 |
| Cognitive distortions | CDQ | Labeling | 2 |
| Cognitive distortions | CDQ | Personalization and Blame | 2 |
| Emotion regulation strategies | CERQ-P | Self-Blame | 4 |
| Emotion regulation strategies | CERQ-P | Rumination | 4 |
| Emotion regulation strategies | CERQ-P | Putting into Perspective | 4 |
| Emotion regulation strategies | CERQ-P | Catastrophizing | 4 |
| Emotion regulation strategies | CERQ-P | Positive Reappraisal | 4 |
| Emotion regulation strategies | CERQ-P | Acceptance | 4 |
| Emotion regulation strategies | CERQ-P | Refocus on Planning | 4 |
| Emotion regulation strategies | CERQ-P | Positive Refocusing | 4 |
| Emotion regulation strategies | CERQ-P | Other-Blame | 4 |

**Note. The 27 network nodes correspond to the four psychological domains analyzed in the study. Node labels, instruments, item counts, and score variables are provided for reference.**
