# Supplementary material for: Insomnia and psychological disintegration: Evidence from a transdiagnostic network analysis
Source: PLoS One. 2026 Jul 23;21(7):e0354243. doi: 10.1371/journal.pone.0354243 (PMC13395365; doi:10.1371/journal.pone.0354243)
Supplement: S3 Table — (DOCX) [file pone.0354243.s003.docx]

**S3 Table. Descriptive statistics (means and standard deviations) for study variables by group.**

| **Domain** | **Node label** | **Poor Sleepers (n=169) M (SD)** | **Good Sleepers (n=222) M (SD)** |
| --- | --- | --- | --- |
| Personality traits | Emotional Stability | 8.22 (2.92) | 10.12 (2.67) |
| Personality traits | Openness to Experience | 11.74 (2.70) | 12.22 (2.30) |
| Personality traits | Conscientiousness | 8.66 (2.54) | 10.08 (2.59) |
| Personality traits | Agreeableness | 11.54 (2.54) | 11.96 (2.22) |
| Personality traits | Extraversion | 9.46 (2.96) | 10.01 (2.79) |
| Emotional symptoms | Stress | 9.94 (4.46) | 7.22 (4.43) |
| Emotional symptoms | Anxiety | 6.86 (4.32) | 3.89 (3.30) |
| Emotional symptoms | Depression | 8.05 (4.77) | 5.10 (4.10) |
| Cognitive distortions | All-or-Nothing Thinking | 5.07 (1.64) | 4.56 (1.62) |
| Cognitive distortions | Overgeneralization | 6.54 (1.81) | 5.52 (1.76) |
| Cognitive distortions | Mental Filters | 5.66 (1.98) | 4.85 (1.80) |
| Cognitive distortions | Disqualifying the Positive | 5.44 (2.25) | 4.48 (1.96) |
| Cognitive distortions | Jumping to Conclusions | 5.96 (2.07) | 4.83 (1.86) |
| Cognitive distortions | Magnification and Minimization | 6.51 (1.87) | 5.74 (2.01) |
| Cognitive distortions | Emotional Reasoning | 5.49 (2.13) | 4.47 (1.78) |
| Cognitive distortions | Should Statements | 6.80 (1.80) | 5.92 (1.91) |
| Cognitive distortions | Labeling | 5.21 (2.09) | 4.40 (2.03) |
| Cognitive distortions | Personalization and Blame | 5.70 (2.10) | 4.82 (1.77) |
| Emotion regulation strategies | Self-Blame | 10.69 (3.30) | 10.46 (3.10) |
| Emotion regulation strategies | Rumination | 12.85 (3.83) | 12.41 (3.35) |
| Emotion regulation strategies | Putting into Perspective | 11.70 (3.32) | 11.74 (3.22) |
| Emotion regulation strategies | Catastrophizing | 10.82 (3.15) | 9.33 (3.06) |
| Emotion regulation strategies | Positive Reappraisal | 12.21 (3.69) | 13.69 (4.15) |
| Emotion regulation strategies | Acceptance | 11.89 (3.00) | 11.59 (3.19) |
| Emotion regulation strategies | Refocus on Planning | 12.36 (3.77) | 13.78 (4.20) |
| Emotion regulation strategies | Positive Refocusing | 11.31 (3.45) | 12.05 (3.86) |
| Emotion regulation strategies | Other-Blame | 9.63 (3.04) | 8.41 (2.60) |

**Note. Values are presented as mean (SD). Descriptive statistics are reported separately for poor sleepers (n = 169) and good sleepers (n = 222).**
